# IMPACT OF THE COVID-19 PANDEMIC: HEALTH AND MENTAL HEALTH CAPACITY OF CAREGIVERS AND CARE RECIPIENTS

**DOI:** 10.1093/geroni/igac059.2344

**Published:** 2022-12-20

**Authors:** Christina Miyawaki, Nina Tahija

**Affiliations:** University of Houston, Houston, Texas, United States; The Council on Recovery, Houston, Texas, United States

## Abstract

We developed a depression intervention, Caregiver-Provided Life Review (C-PLR) for people living with dementia and their family caregivers in 2018 and pilot-tested (N=19 caregiver (CG)-care recipient (CR) dyads). Encouraged by the outcomes, in 2020 we expanded the study to long-term care facility (LTCF) residents. Due to the COVID-19 pandemic, we adapted the in-person mode to virtual and recruited CGs who provided care for >8 hours/week and CRs who were community-dwellers or LTCF residents with mild depression and dementia. Between August 2020 and December 2021, we contacted 195 LTCFs, 29 senior/community centers, and 148 community-dwellers and recruited 12 dyads nationwide. CGs were on average 54 years old, working (67%), college-educated (83%), female (92%), and in good-excellent health (75%). CRs were 83 years old (mean), single (58%), female (92%), and in fair health (50%). CRs’ depression score significantly improved (p=0.0004), however, CG’s burden and positive aspects of caregiving scores worsened (p=0.02). Amid the pandemic, LTCF closures and staff turnover made recruitment challenging. Despite a small sample, we found CRs enjoy the LRs alleviating depressive symptoms. However, CGs experienced more burden, which could be due to COVID fatigue and the LR as an additional responsibility. These results reminded us of the COVID impact and adjusted the C-PLR model further. Instead of a weekly visit, each dyad can meet at its own pace. We developed the C-PLR to improve the health of the dyads. Thus, we must consider both CGs’ and CRs’ health capacity because CR’s wellbeing is dependent on that of CGs.

